# Supplementary material for: Structural and mechanistic insights into Streptococcus pneumoniae NADPH oxidase
Source: Nat Struct Mol Biol. 2024 Jul 22;31(11):1769–77. doi: 10.1038/s41594-024-01348-w (PMC11564096; doi:10.1038/s41594-024-01348-w)

nature structural & molecular biology

Supplementary information https://doi.org/10.1038/s41594-024-01348-w

Structural and mechanistic insights into *Streptococcus pneumoniae* NADPH oxidase

In the format provided by the authors and unedited

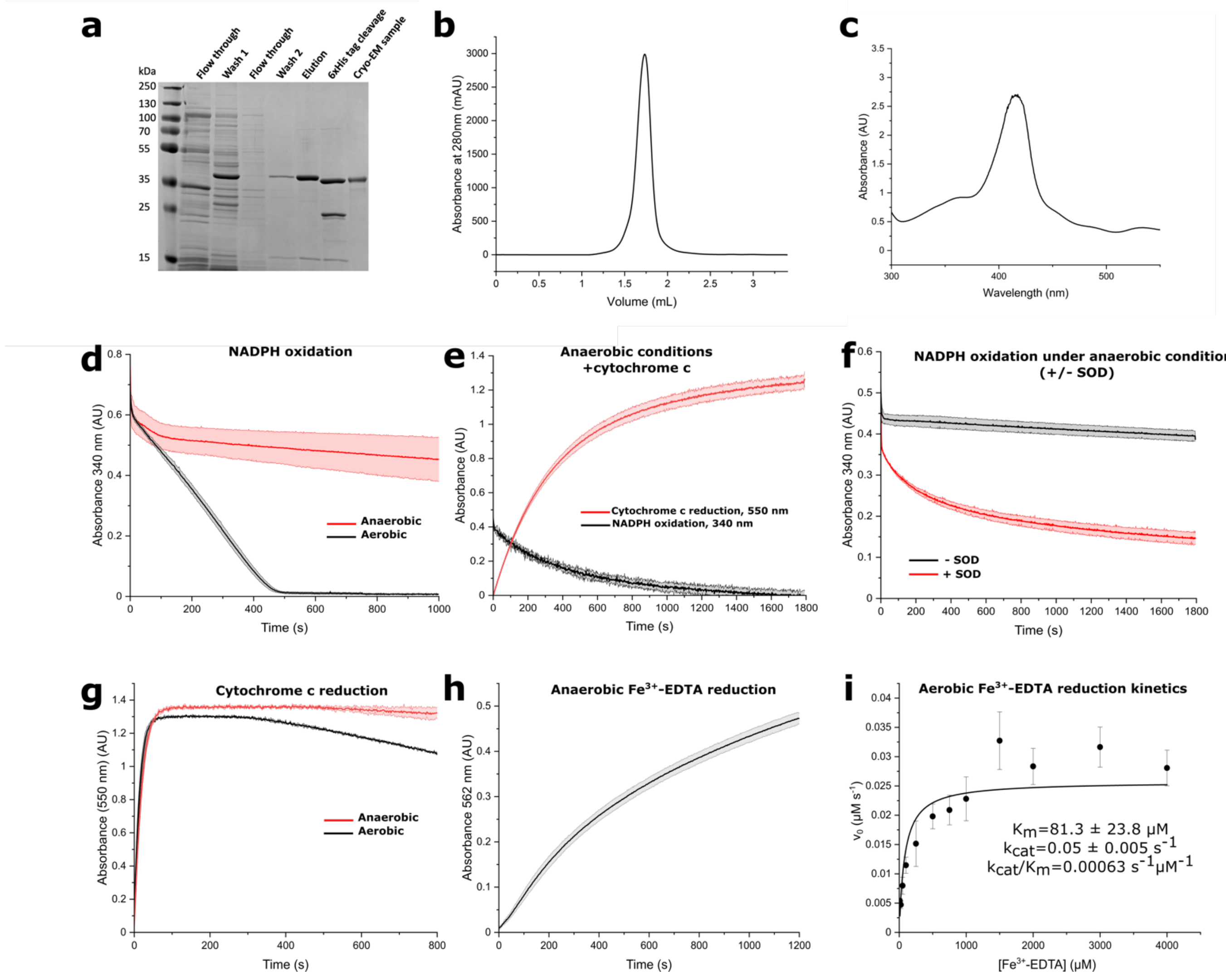

Supplementary Fig. 1 | SpNOX for structural studies was highly pure and active

a, Analysis of the relevant fractions of SpNOX purification by Coomassie blue staining after 12% SDS-PAGE under reducing conditions. **b,** Representative size-exclusion chromatography (SEC) profile of SpNOX in LMNG micelles before cryo-EM grid freezing. **c,** UV-vis absorbance spectrum of SpNOX displaying the Soret peak at 414 nm. Purification experiments of SpNOX in **(a-c)** were performed independently at least three times **d,** Directly monitoring the NADPH concentration provides a measure of cytochrome c-independent activity, which is high in the presence of oxygen (black trace), and only shows initial activity before stalling in anaerobic conditions (red trace). **e,** Anaerobic NADPH oxidation (340 nm, black trace) and cytochrome c reduction activity (550 nm, red trace) of SpNOX showing (direct) cytochrome c reduction in the absence of oxygen. **f,** Anaerobic NADPH oxidation activity of SpNOX in the presence of superoxide dismutase (SOD) showing continued NADPH oxidation in the absence of oxygen. **g,** Cytochrome c reduction activity of SpNOX shows a highly similar initial rate under aerobic (black) and anaerobic (red) conditions. **h,** Anaerobic Fe(III)-EDTA reduction followed by Fe(II)-Ferrozine absorbance (562 nm) shows direct electron transfer to Fe(III) forming Fe(II). **i,** Michaelis-Menten kinetics of Fe(III)-EDTA reduction by SpNOX under aerobic conditions. Mean values of three technical replicates are plotted and SD are indicated. Data for individual replicates are available in the source data file.

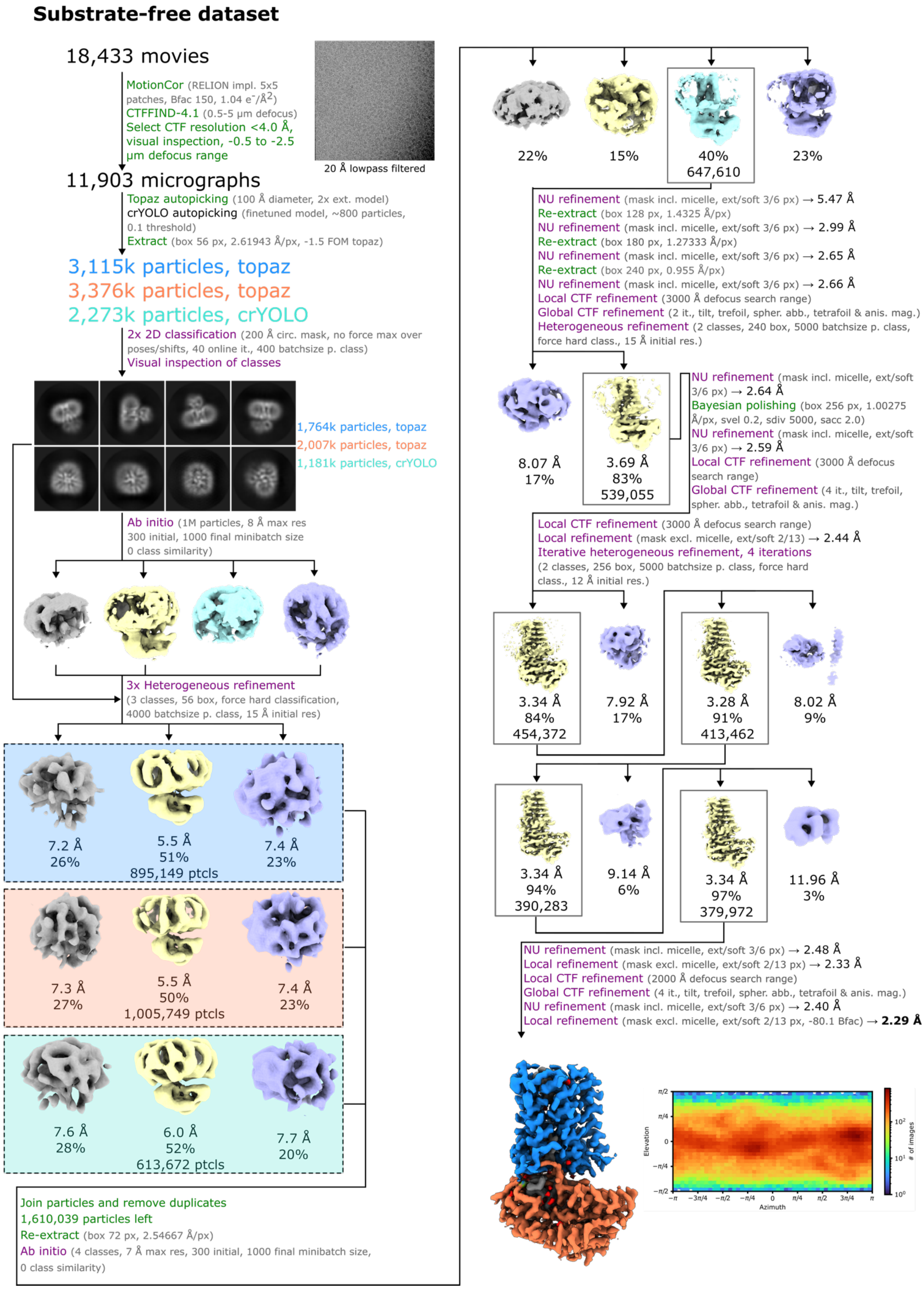

Supplementary Fig. 2 | Cryo-EM processing workflow for substrate-free SpNOX reconstruction

Processes run in RELION are colored green, while processes run in cryoSPARC are colored purple. Abbreviations used: External (ext.), pixel (px), per (p.), resolution (res.), particles (ptcls), including (incl.), extension (ext), soft-edge (soft), classification (class.), iterations (it.), excluding (excl.), spherical aberration (spher. abb.), anisotropic magnification (anis. mag.) and B-factor (Bfac).

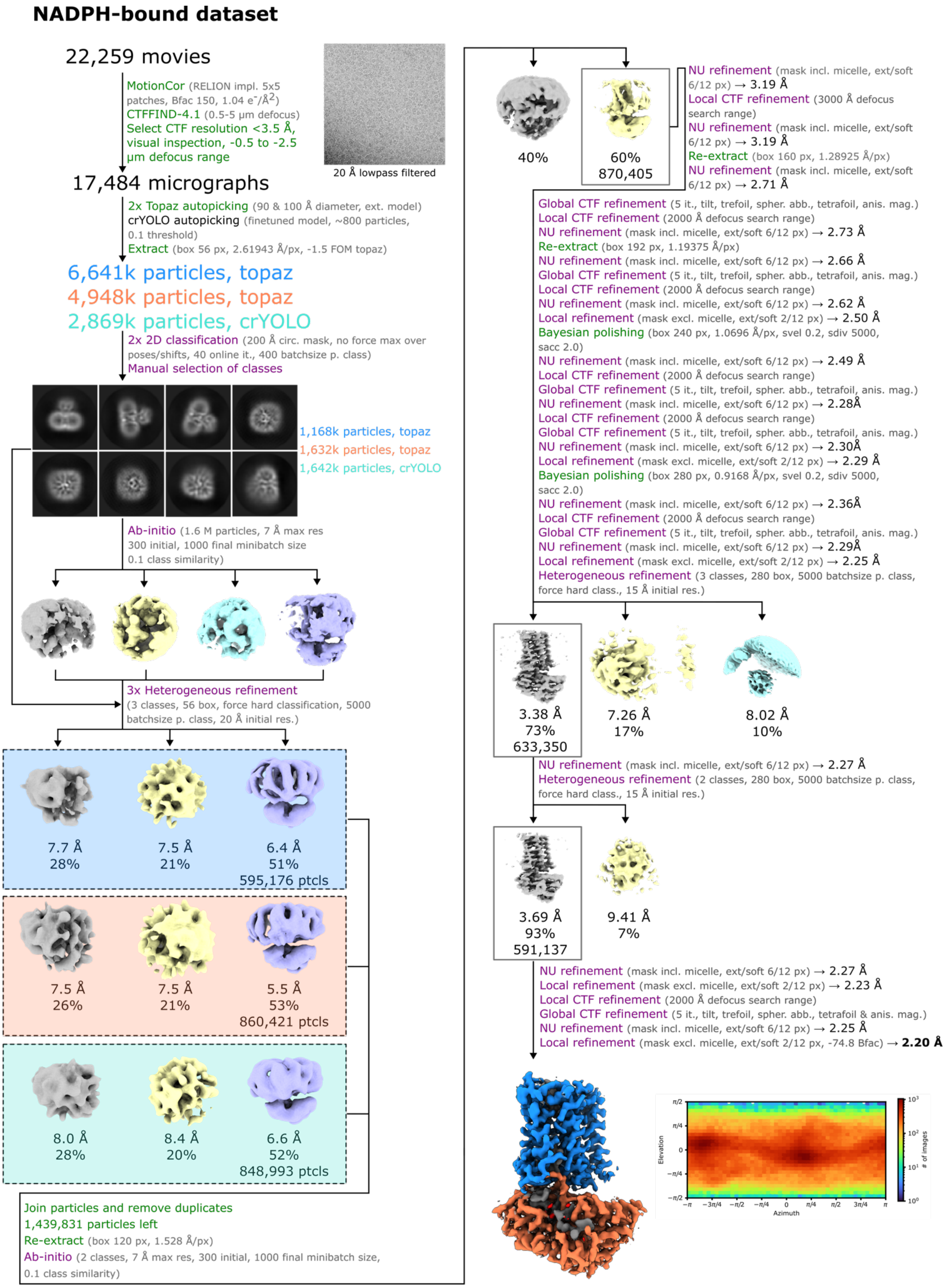

Supplementary Fig. 3 | Cryo-EM processing workflow for NADPH-bound SpNOX reconstruction
Processes run in RELION are colored green, while processes run in cryoSPARC are colored purple. Abbreviations used: External (ext.), pixel (px), per (p.), resolution (res.), particles (ptcls), including (incl.), extension (ext), soft-edge (soft), classification (class.), iterations (it.), excluding (excl.), spherical aberration (spher. abb.), anisotropic magnification (anis. mag.) and B-factor (Bfac).

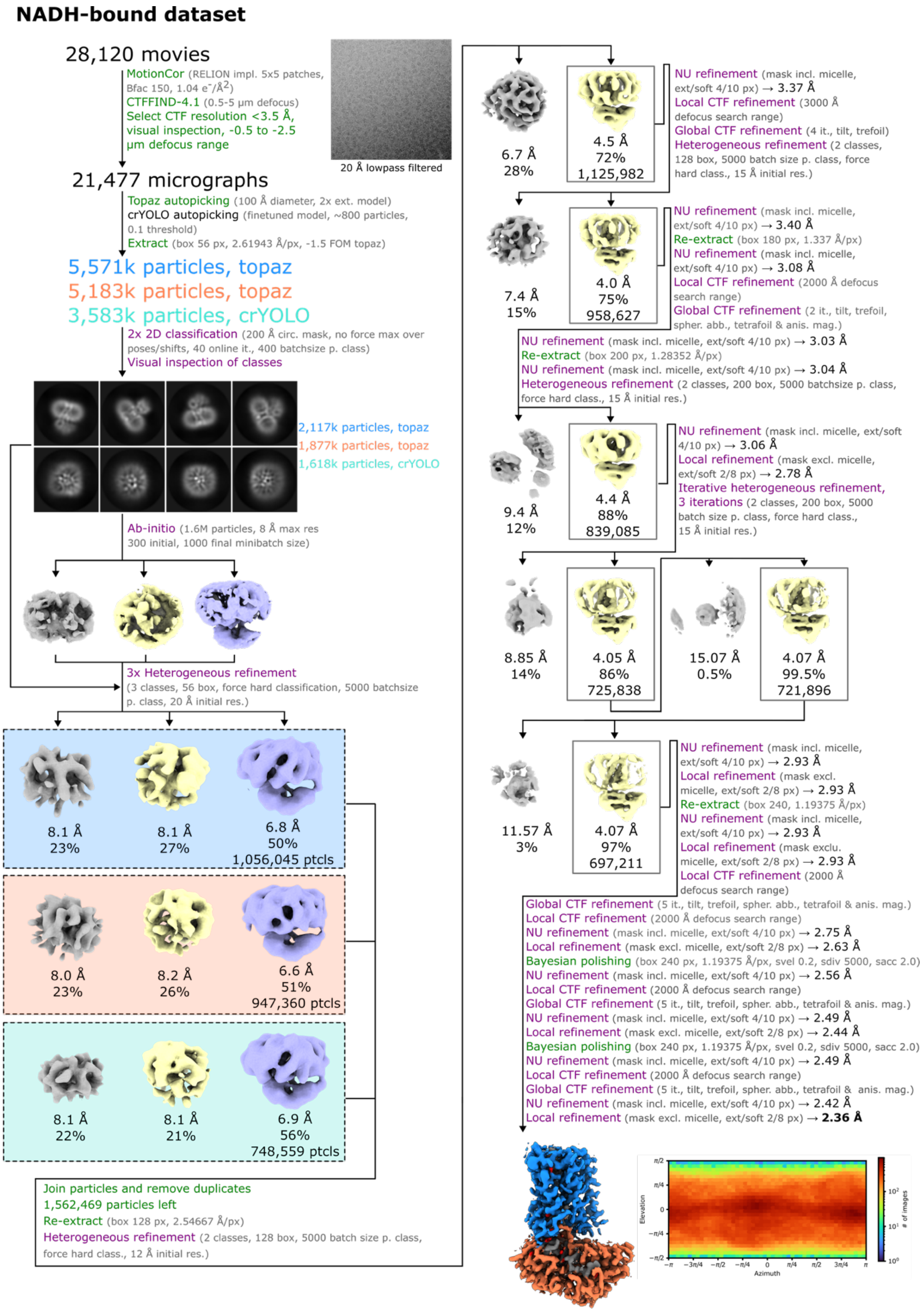

Supplementary Fig. 4 | Cryo-EM processing workflow for NADH-bound SpNOX reconstruction

Processes run in RELION are colored green, while processes run in cryoSPARC are colored purple. Abbreviations used: External (ext.), pixel (px), per (p.), resolution (res), particles (ptcls), including (incl.), extension (ext), soft-edge (soft), classification (class.), iterations (it.), excluding (excl.), spherical aberration (spher. abb.), anisotropic magnification (anis. mag.) and B-factor (Bfac).

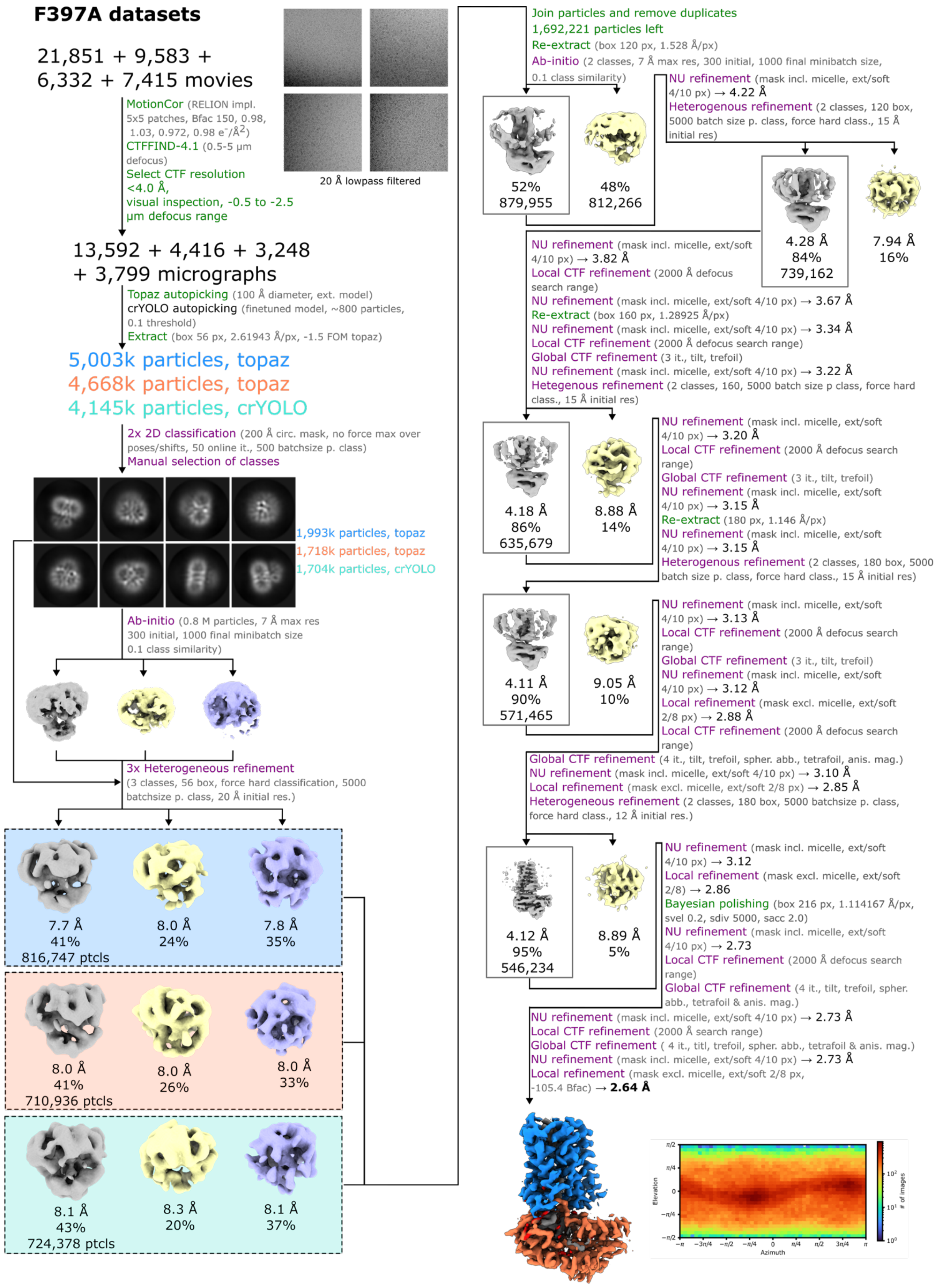

Supplementary Fig. 5 | Cryo-EM processing workflow for NADPH-bound Phe397Ala SpNOX reconstruction
Processes run in RELION are colored green, while processes run in cryoSPARC are colored purple. Abbreviations used: External (ext.), pixel (px), per (p.), resolution (res.), particles (ptcls), including (incl.), extension (ext), soft-edge (soft), classification (class.), iterations (it.), excluding (excl.), spherical aberration (spher. abb.), anisotropic magnification (anis. mag.) and B-factor (Bfac).

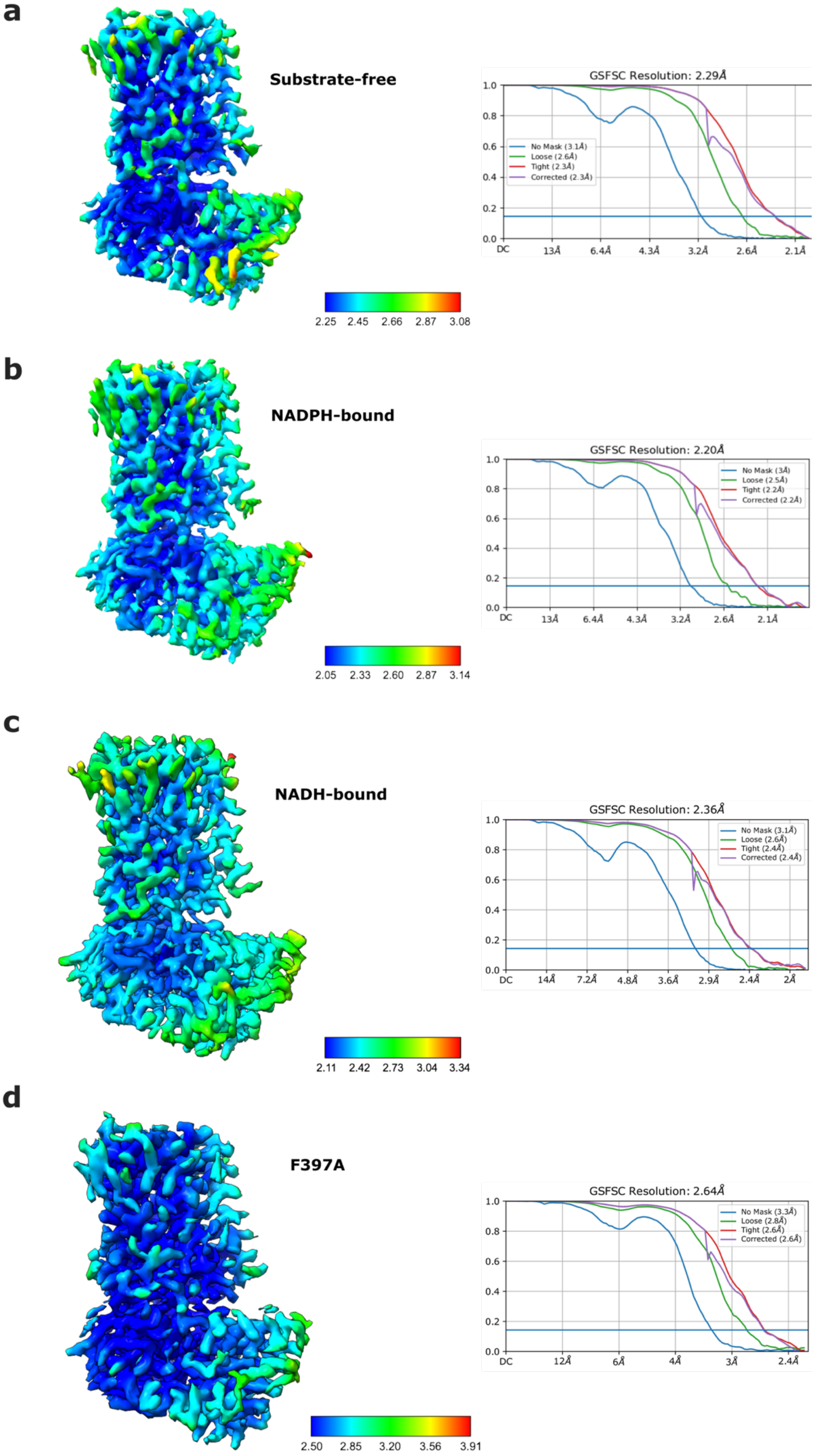

Supplementary Fig. 6 | Local resolution and FSC curves of the cryo-EM reconstructions

a-d, Left: Density maps of the SpNOX reconstructions colored by local resolution. Right: Gold-standard Fourier Shell Correlation (FSC) curves of the 3D reconstructions indicating the resolution at FSC=0.143.

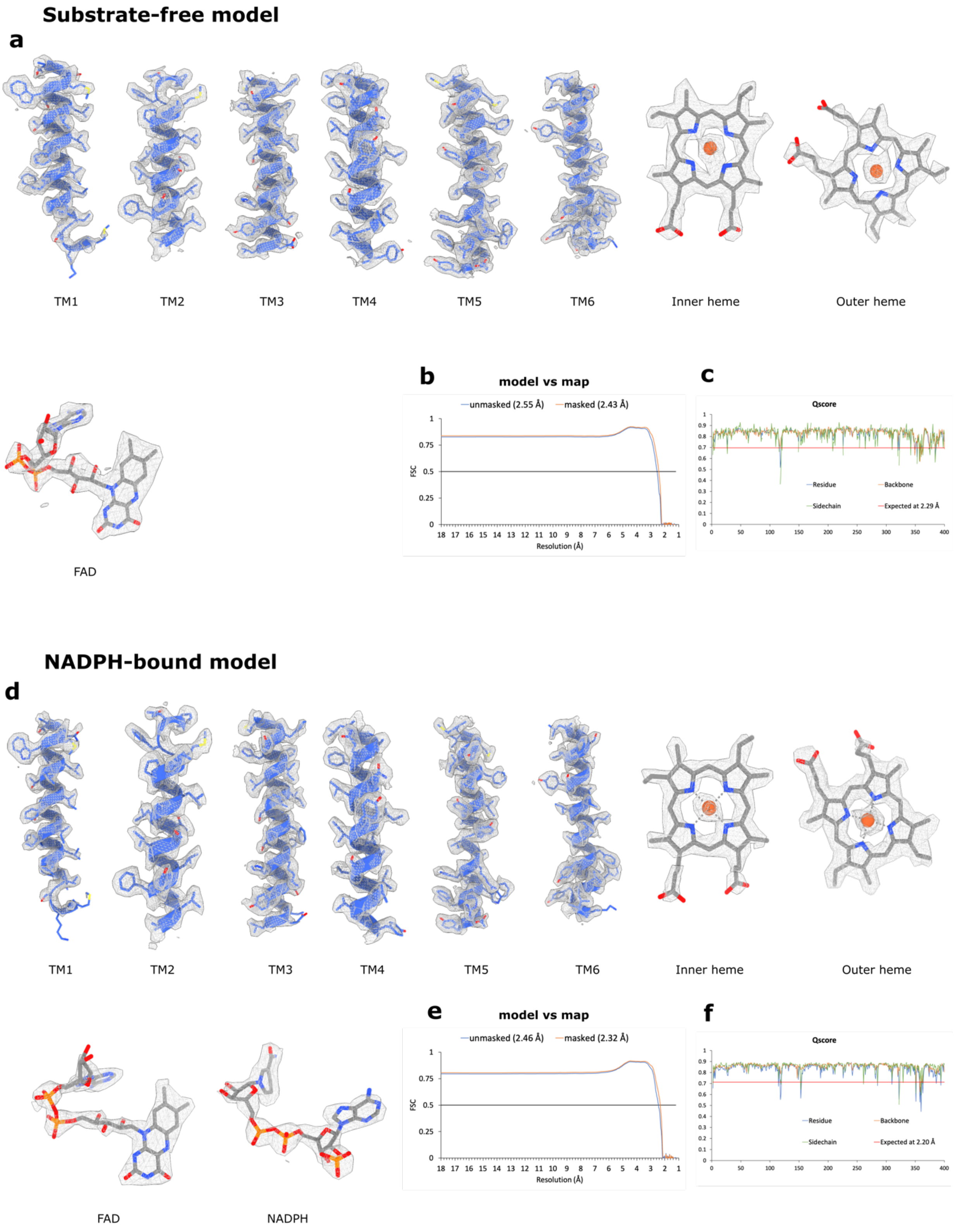
Substrate-free model
a
TM1
TM2
TM3
TM4
TM5
TM6
Inner heme
Outer heme
FAD
b
model vs map
unmasked (2.55 Å)
masked (2.43 Å)
FSC
Resolution (Å)
c
Qscore
Residue
Backbone
Sidechain
Expected at 2.29 Å
NADPH-bound model
d
TM1
TM2
TM3
TM4
TM5
TM6
Inner heme
Outer heme
FAD
NADPH
e
model vs map
unmasked (2.46 Å)
masked (2.32 Å)
FSC
Resolution (Å)
f
Qscore
Residue
Backbone
Sidechain
Expected at 2.20 Å

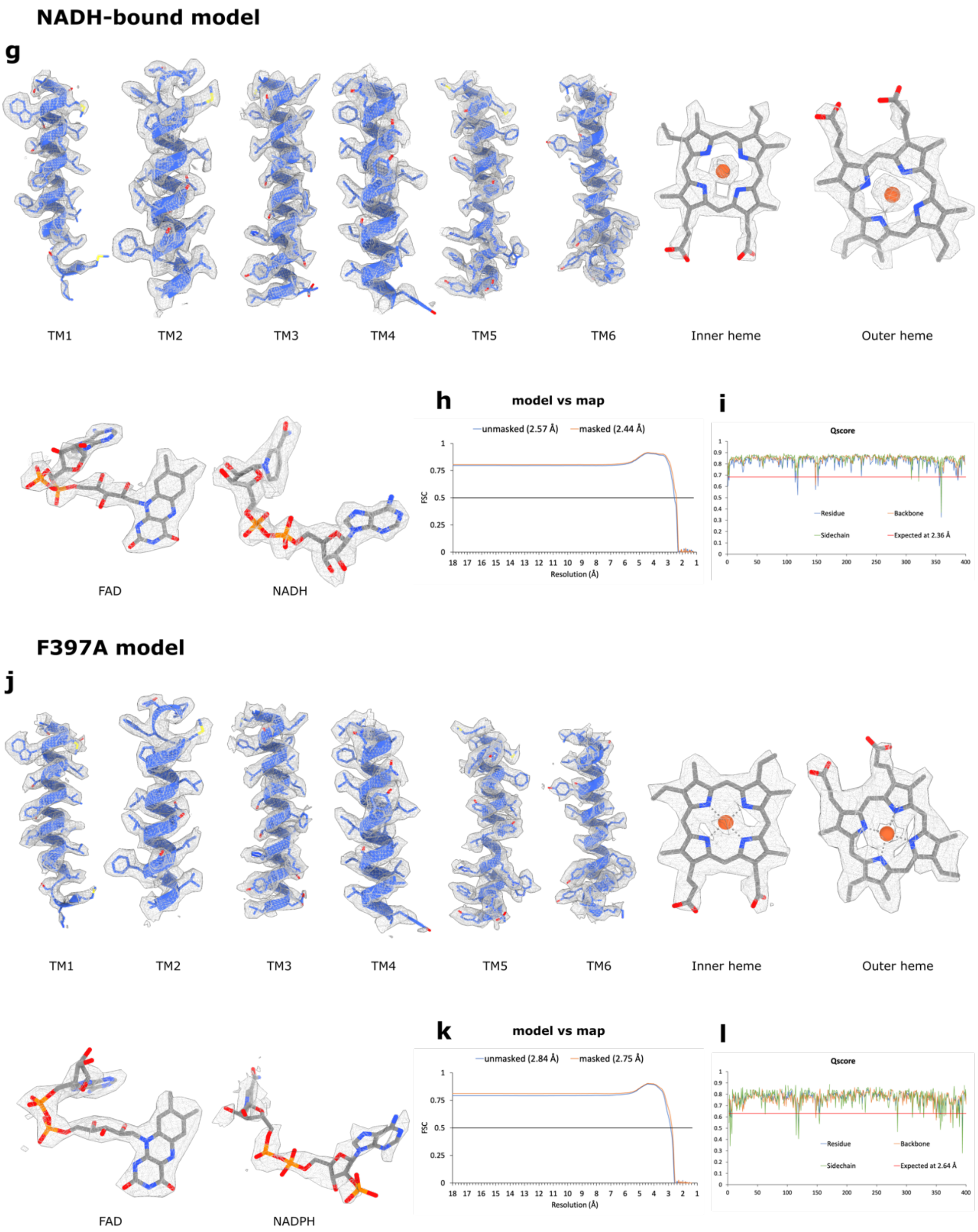

Supplementary Fig. 7 | SpNOX model vs map validation

a, Density fit of the transmembrane helices and cofactors of the substrate-free model. **b,** Model vs map FSC curve. **c,** Q-score of the residue average, backbone, side chain and expected value for map resolution. **d,** Density fit of the transmembrane helices and cofactors of the NADPH-bound model. **e,** Model vs map FSC curve. **f,** Q-score of the residue average, backbone, side chain and expected value for map resolution. **g,** Density fit of the transmembrane helices and cofactors of the NADH-bound model. **h,** Model vs map FSC curve. **i,** Q-score of the residue average, backbone, side chain and expected value for map resolution. **j,** Density fit of the transmembrane helices and cofactors of the Phe397Ala model. **k,** Model vs map FSC curve. **l,** Q-score of the residue average, backbone, side chain and expected value for map resolution.

a

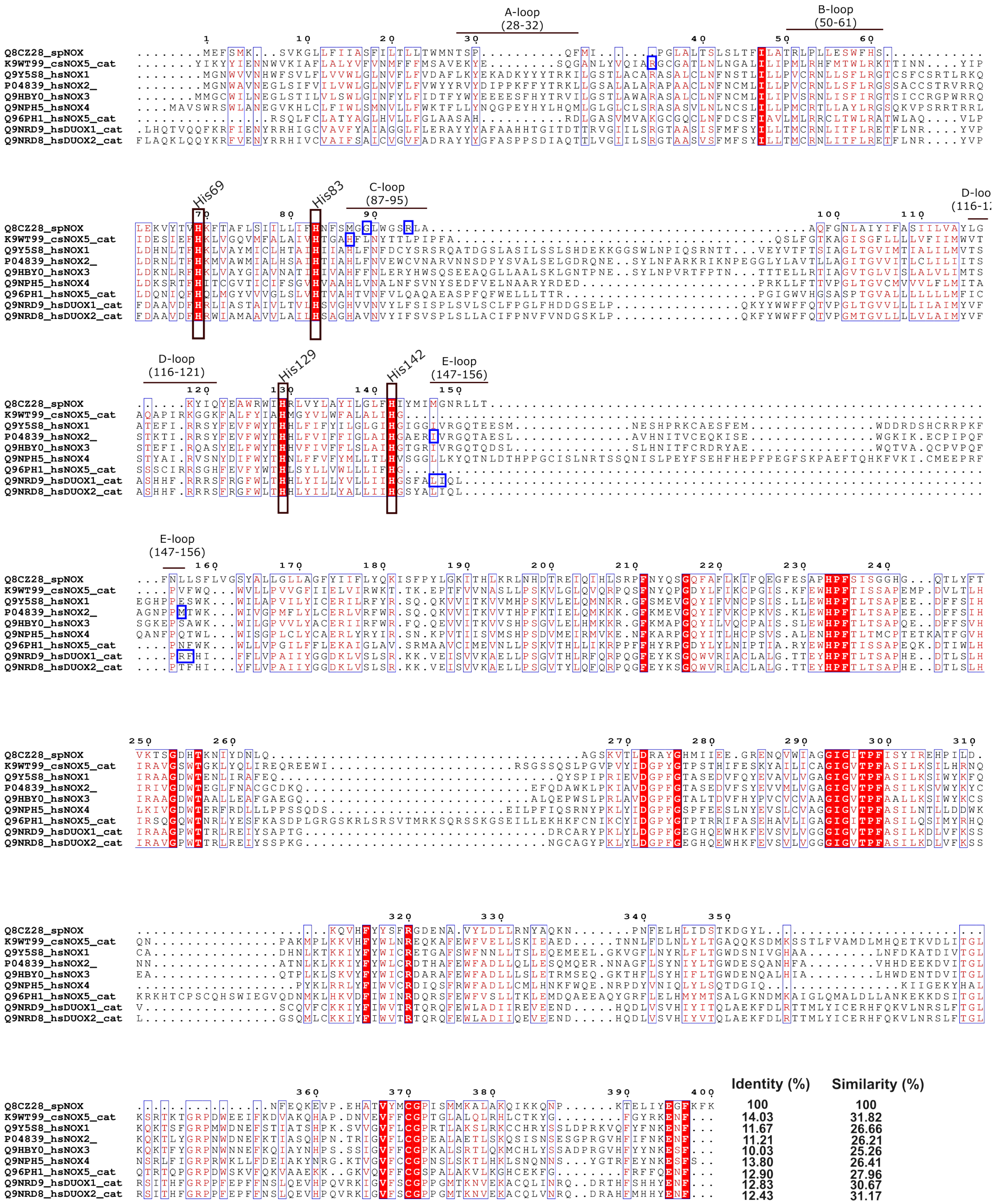
A-loop (28-32)
B-loop (50-61)
C-loop (87-95)
D-loop (116-121)
E-loop (147-156)
His69
His83
His129
His142
Q8CZ28_spNOX
K9WT99_csNOX5_cat
Q9Y5S8_hsNOX1
P04839_hsNOX2_
Q9HBY0_hsNOX3
Q9NPH5_hsNOX4
Q96PH1_hsNOX5_cat
Q9NRD9_hsDUOX1_cat
Q9NRD8_hsDUOX2_cat
Identity (%)
Similarity (%)
100
100
14.03
31.82
11.67
26.66
11.21
26.21
10.03
25.26
13.80
26.41
12.90
27.96
12.83
30.67
12.43
31.17

b

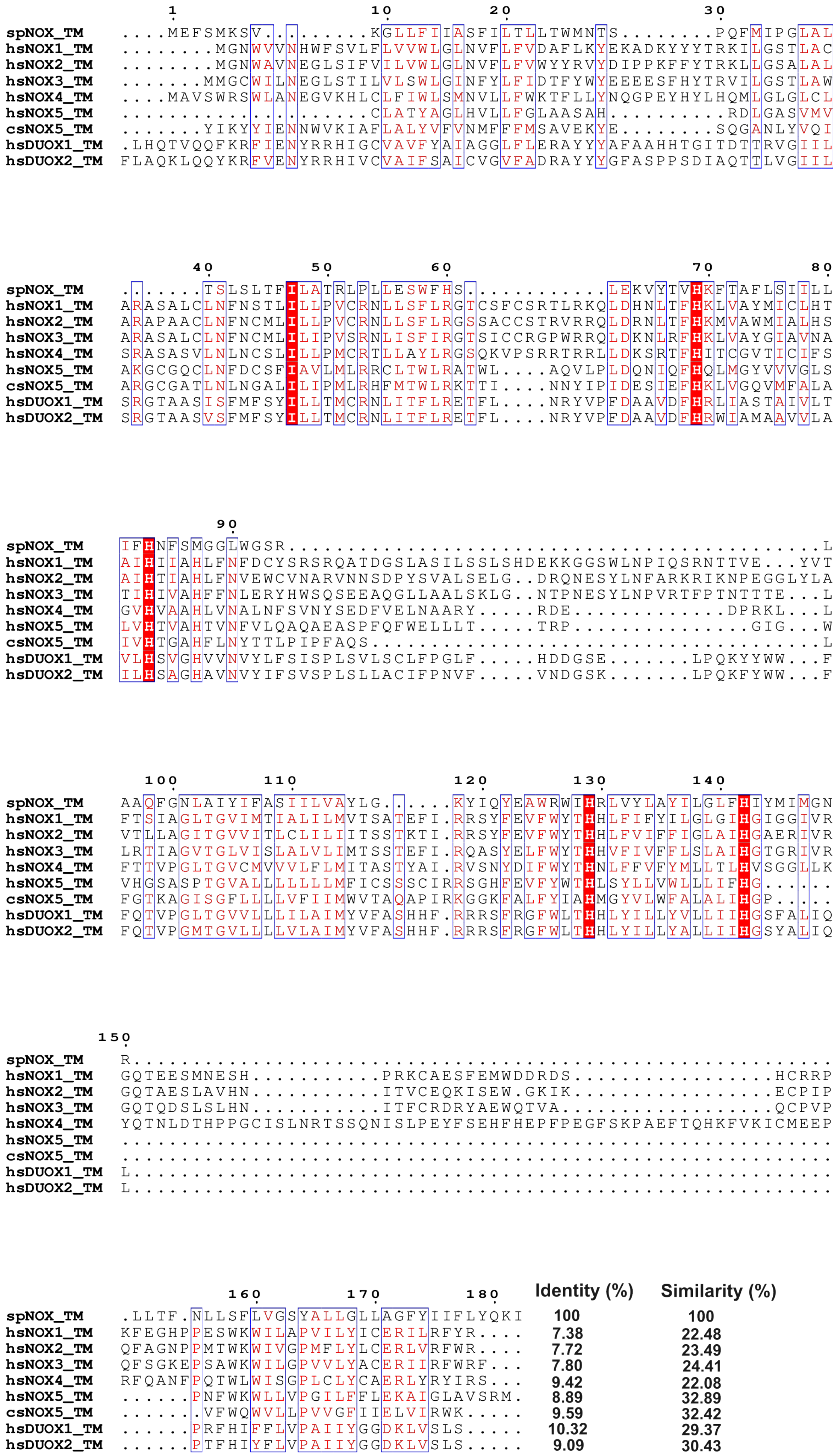
1 10 20 30
spNOX_TM ....MEFSMKSV.........KGLLFIIASFILTLLTWMNTS........PQFMIPGLAL
hsNOX1_TM ........MGNWVVNHWFSVLFLVVWLGLNVFLFVDAFLKYEKADKYYYTRKILGSTLAC
hsNOX2_TM ........MGNWAVNEGLSIFVILVWLGLNVFLFVWYYRVYDIPPKFFYTRKLLGSALAL
hsNOX3_TM .......MMGCWILNEGLSTILVLSWLGINFYLFIDTFYWYEEEESFHYTRVILGSTLAW
hsNOX4_TM ....MAVSWRSWLANEGVKHLCLFIWLSMNVLLFWKTFLLYNQGPEYHYLHQMLGLGLCL
hsNOX5_TM ......................CLATYAGLHVLLFGLAASAH..........RDLGASVMV
csNOX5_TM .......YIKYYIENNWVKIAFLALYVFVNMFFFMSAVEKYE.........SQGANLYVQI
hsDUOX1_TM .LHQTVQQFKRFIENYRRHIGCVAVFYAIAGGLFLERAYYYAFAAHHTGITDTTRVGIIL
hsDUOX2_TM FLAQKLQQYKRFVENYRRHIVCVAIFSAICVGVFADRAYYYGFASPPSDIAQTTLVGIIL
40 50 60 70 80
spNOX_TM ........TSLSLTFILATRLPLLESWFHS............LEKVYTVHKFTAFLSIILL
hsNOX1_TM ARASALCLNFNSTLILLPVCRNLLSFLRGTCSFCSRTLRKQLDHNLTFHKLVAYMICLHT
hsNOX2_TM ARAPAACLNFNCMLILLPVCRNLLSFLRGSSACCSTRVRRQLDRNLTFHKMVAWMIALHS
hsNOX3_TM ARASALCLNFNCMLILIPVSRNLISFIRGTSICCRGPWRRQLDKNLRFHKLVAYGIAVNA
hsNOX4_TM SRASASVLNLNCSLILLPMCRTLLAYLRGSQKVPSRRTRRLLDKSRTFHITCGVTICIFS
hsNOX5_TM AKGCGQCLNFDCSFIAVLMLRRCLTWLRATWL....AQVLPLDQNIQFHQLMGYVVVGLS
csNOX5_TM ARGCGATLNLNGALILIPMLRHFMTWLRKTTI....NNYIPIDESIEFHKLVGQVMFALA
hsDUOX1_TM SRGTAASISFMFSYILLTMCRNLITFLRETFL....NRYVPFDAAVDFHRLIASTAIVLT
hsDUOX2_TM SRGTAASVSFMFSYILLTMCRNLITFLRETFL....NRYVPFDAAVDFHRWIAMAAVVLA
90
spNOX_TM IFHNFSMGGLWGSR..L
hsNOX1_TM AIHIIAHLFNFDCYSRSRQATDGSLASILSSLSHDEKKGGSWLNPIQSRNTTVE...YVT
hsNOX2_TM AIHTIAHLFNVEWCVNARVNNSDPYSVALSELG.DRQNESYLNFARKRIKNPEGGLYLA
hsNOX3_TM TIHIVAHFFNLERYHWSQSEEAQGLLAALSKLG.NTPNESYLNPVRTFPTNTTTE...L
hsNOX4_TM GVHVAAHLVNALNFSVNYSEDFVELNAARY.....RDE.............DPRKL...L
hsNOX5_TM LVHTVAHTVNFVLQAQAEASPFQFWELLLT.....TRP...............GIG...W
csNOX5_TM IVHTGAHFLNYTTLPIPFAQS.......................................L
hsDUOX1_TM VLHSVGHVVNVYLFSISPLSVLSCLFPGLF.....HDDGSE.......LPQKYYWW...F
hsDUOX2_TM ILHSAGHAVNVYIFSVSPLSLLACIFPNVF.....VNDGSK.......LPQKFYWW...F
100 110 120 130 140
spNOX_TM AAQFGNLAIYIFASIILVAYLG......KYIQYEAWRWIHRLVYLAYILGLFHIYMIMGN
hsNOX1_TM FTSIAGLTGVIMTIALILMVTSATEFI.RRSYFEVFWYTHHLFIFYILGLGIHGIGGIVR
hsNOX2_TM VTLLAGITGVVITLCLILIITSSTKTI.RRSYFEVFWYTHHLFVIFFIGLAIHGAERIVR
hsNOX3_TM LRTIAGVTGLVISLALVLIMTSSTEFI.RQASYELFWYTHHVFIVFFLSLAIHGTGRIVR
hsNOX4_TM FTTVPGLTGVCMVVVLFLMITASTYAI.RVSNYDIFWYTHNLFFVFYMLLTLHVSGGLLK
hsNOX5_TM VHGSASPTGVALLLLLLMFICSSSCIRRSGHFEVFYWTHLSYLLVWLLLIFHG......
csNOX5_TM FGTKAGISGFLLLLVFIIMWVTAQAPIRKGGKFALFYIAHMGYVLWFALALIHGP.....
hsDUOX1_TM FQTVPGLTGVVLLLILAIMYVFASHHF.RRRSFRGFWLTHHLYILLYVLLIIHGSFALIQ
hsDUOX2_TM FQTVPGMTGVLLLLVLAIMYVFASHHF.RRRSFRGFWLTHHLYILLYALLIIHGSYALIQ
150
spNOX_TM R...
hsNOX1_TM GQTEESMNESH...........PRKCAESFEMWDDRDS.................HCRRP
hsNOX2_TM GQTAESLAVHN...........ITVCEQKISEW.GKIK.................ECPIP
hsNOX3_TM GQTQDSLSLHN...........ITFCRDRYAEWQTVA..................QCPVP
hsNOX4_TM YQTNLDTHPPGCISLNRTSSQNISLPEYFSEHFHEPFPEGFSKPAEFTQHKFVKICMEEP
hsNOX5_TM ....
csNOX5_TM ....
hsDUOX1_TM L...
hsDUOX2_TM L...
160 170 180 Identity (%) Similarity (%)
spNOX_TM .LLTF.NLLSFLVGSYALLGLLAGFYIIFLYQKI 100 100
hsNOX1_TM KFEGHPPESWKWILAPVILYICERILRFYR.... 7.38 22.48
hsNOX2_TM QFAGNPPMTWKWIVGPMFLYLCERLVRFWR.... 7.72 23.49
hsNOX3_TM QFSGKEPSAWKWILGPVVLYACERIIRFWRF... 7.80 24.41
hsNOX4_TM RFQANFPQTWLWISGPLCLYCAERLYRYIRS... 9.42 22.08
hsNOX5_TM ......PNFWKWLLVPGILFFLEKAIGLAVSRM. 8.89 32.89
csNOX5_TM .......VFWQWVLLPVVGFIIELVIRWK..... 9.59 32.42
hsDUOX1_TM ......PRFHIFFLVPAIIYGGDKLVSLS..... 10.32 29.37
hsDUOX2_TM ......PTFHIYFLVPAIIYGGDKLVSLS..... 9.09 30.43

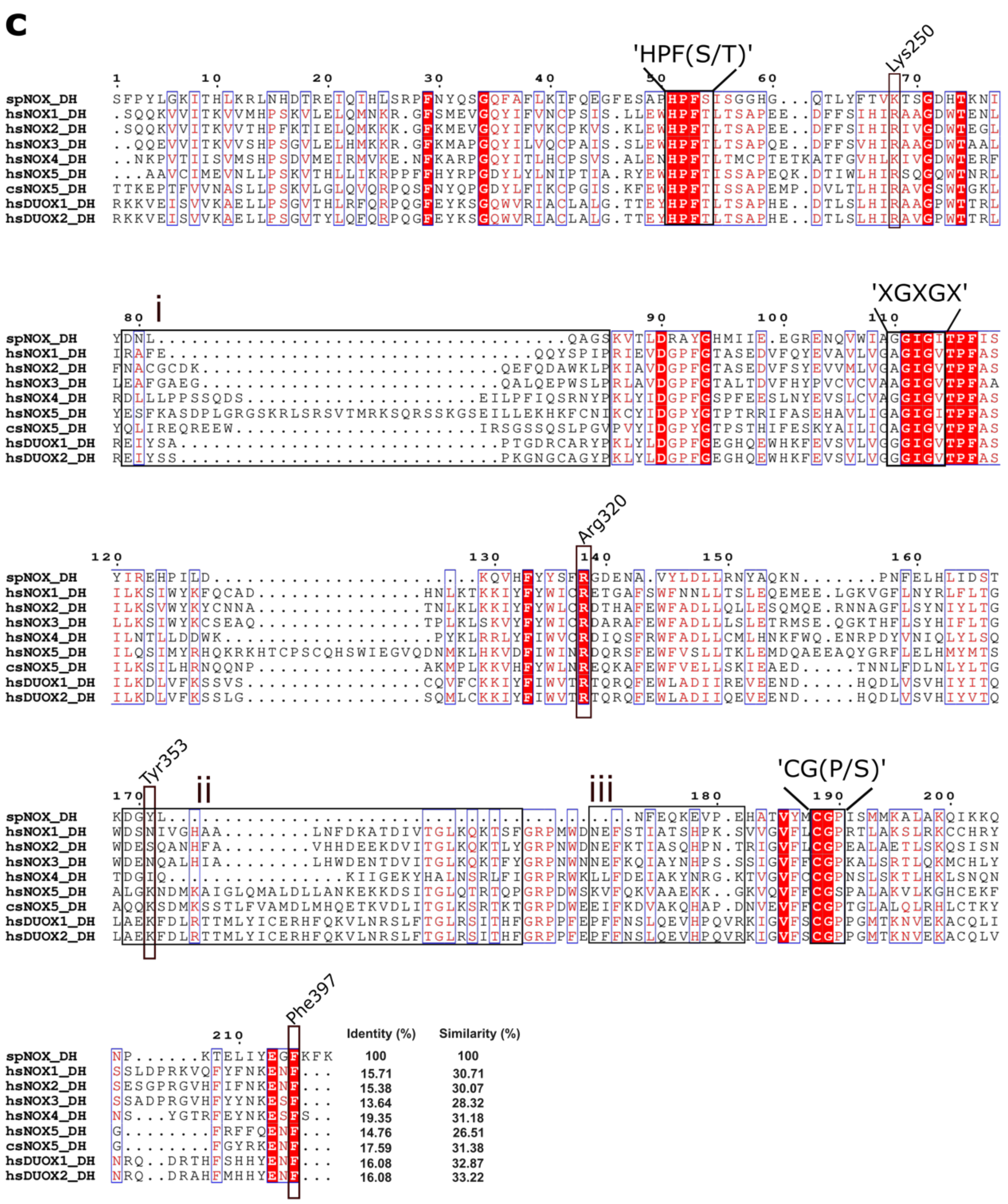

Supplementary Fig. 8 | Sequence alignment of SpNOX with human and cyanobacterial NOXs

All sequence identity values were calculated with respect to SpNOX. **a,** Sequence alignment of full-length SpNOX (Q8CZ28) with the catalytic subunits of human (*Homo sapiens*, hs) NOX 1 (Q9Y5S8), NOX2 (P04839), NOX3 (Q9HBY0) and NOX4 (Q9NPH5); and the catalytic core of human NOX5 (Q96PH1, 236-765), human DUOX1 (Q9NRD9, 1027-1551), human DUOX2 (Q9NRD8, 1023-1548) and *Cylindrospermum stagnale* csNOX5 (K9WT99, 210-693). Heme-coordinating histidines (His69, His83, His129 and His142) and the amino acids of the extracellular (A, C and E) and intracellular (B and D) loops of SpNOX are highlighted. The amino acids interacting with the propionate groups of the outer heme are boxed in blue. **b**, Sequence alignment of SpNOX TM domain (1-182) with the TM domains of human NOX1 (1-290), NOX2 (1-290), NOX3 (1-289), NOX4 (1-305), NOX5 (236-443), DUOX1 (1027-1269), DUOX2 (1023-1266) and csNOX5 (210-409). **c**, Sequence alignment of SpNOX DH domain (183-400) with the DH domains of human NOX1 (291-564), NOX2 (291-570), NOX3 (290-568), NOX4 (306-578), NOX5 (444-765), DUOX1 (1270-1551), DUOX2 (1268-1548) and csNOX5 (410-693). The additional eukaryotic NOX sequences at the DH domain (i, ii and iii as displayed in Supplementary Fig. 9) are boxed; consensus sequences 'HPF(S/T)', 'XGXGX' and 'CG(P/S)', regulatory Phe397 and the amino acids involved in NAD(P)H-binding Lys250, Arg320, Ser348 and Tyr353 are highlighted.

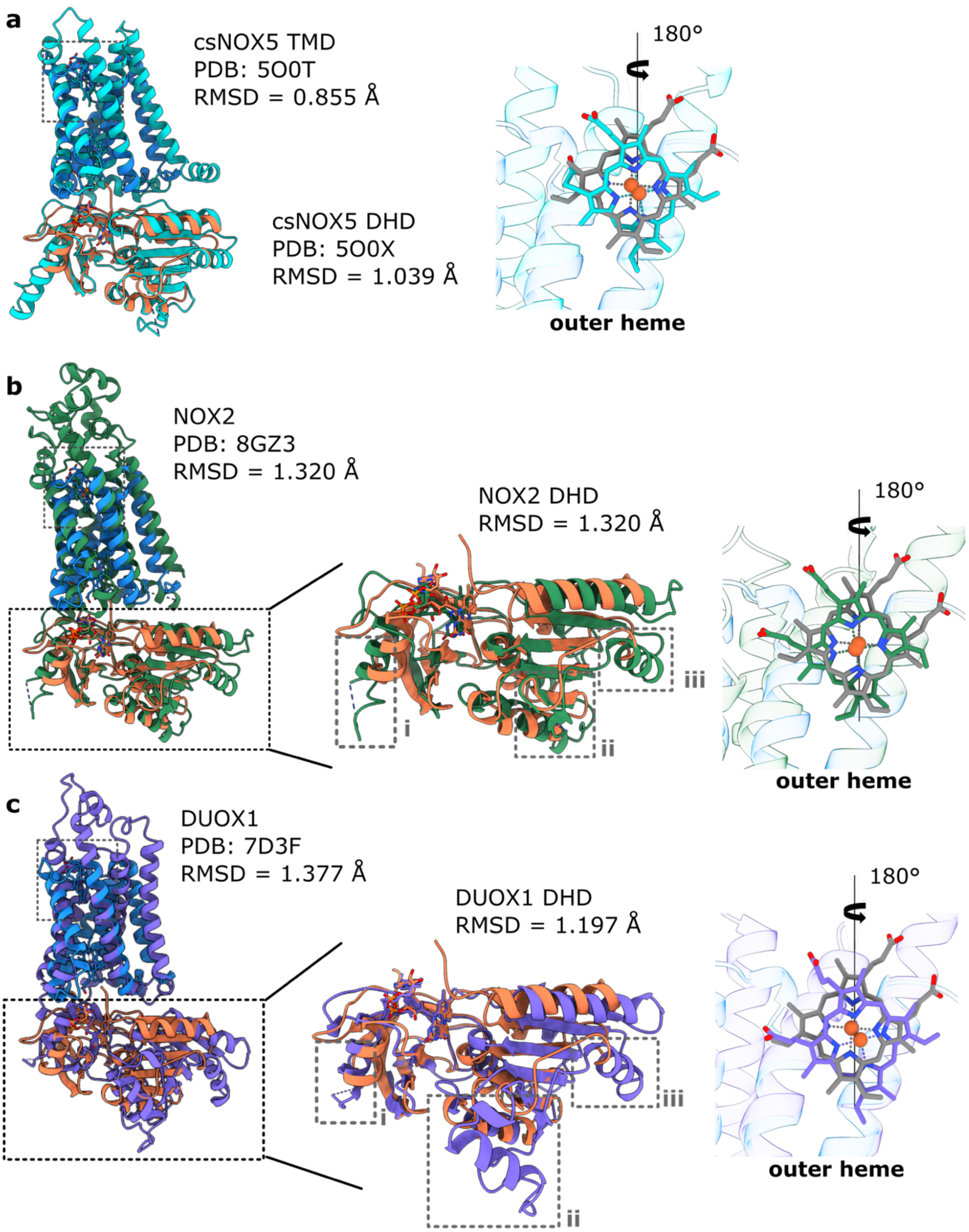

Supplementary Fig. 9 | Structural comparison of SpNOX with human and cyanobacterial NOXs

a, Structure alignment of the csNOX5 TM (PDB: 5O0T) and DH (PDB: 5O0X)[1] domains with SpNOX. RMSD values are given for 40 and 131 residue pairs for the TM and DH domains, respectively. **b,** Structure alignment of full-length NOX2 (PDB: 8GZ3)[2] and DH domain of NOX2 with SpNOX. RMSD values are given for 87 and 78 residue pairs for the full length and DH domain alignments, respectively. **c,** Structure alignment of the catalytic core of human DUOX1 (PDB: 7D3F) [3] and DH domain of human DUOX1 with SpNOX. RMSD values are given for 39 and 88 residue pairs for the full length and DH domain alignments, respectively. Additional structural elements absent in SpNOX but present in NOX2 or DUOX1 are indicated by grey boxes. The outer heme of SpNOX is flipped ~180° with respect to the outer hemes of DUOX1, NOX2 and csNOX5 (right panels).

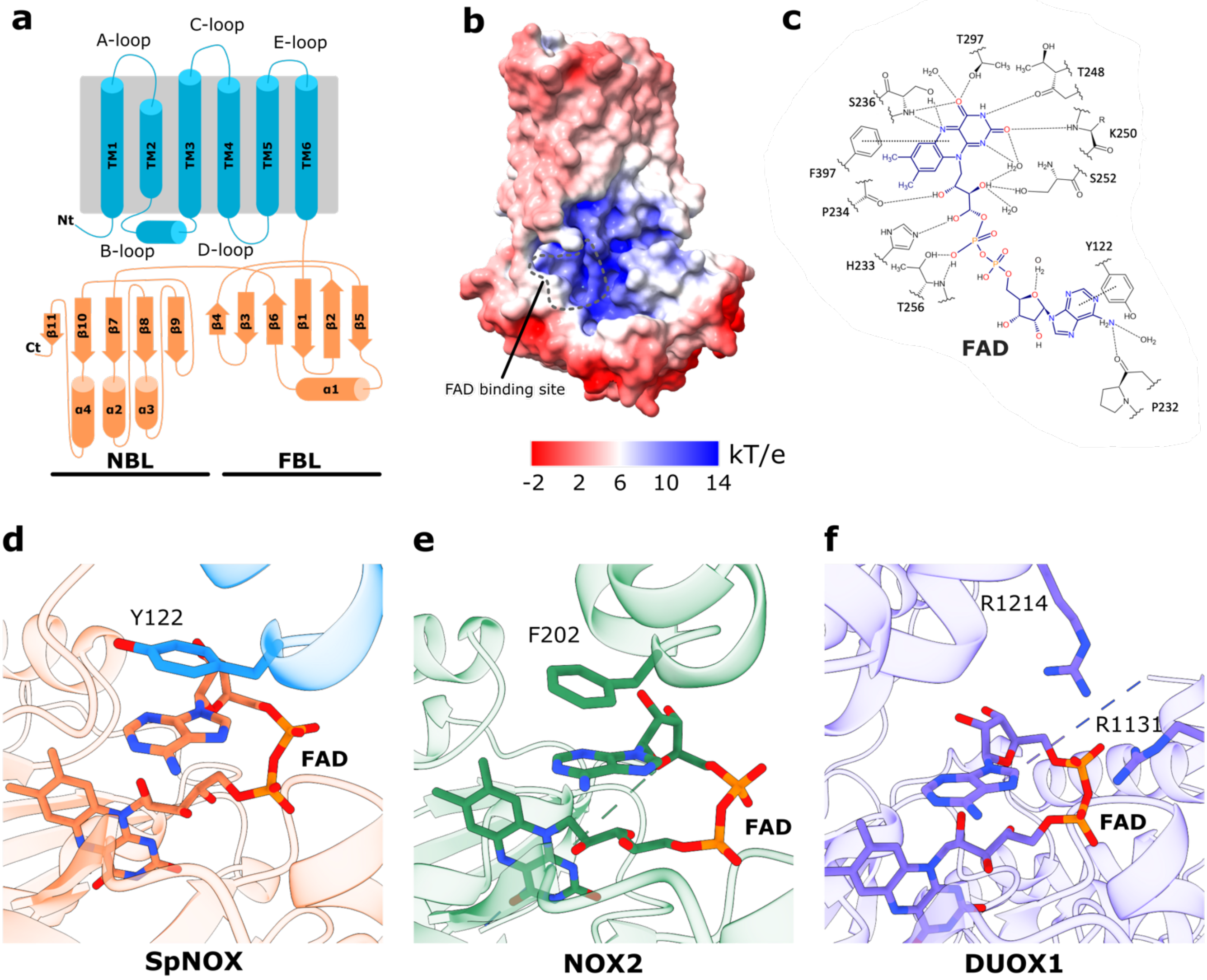

Supplementary Fig. 10 | Structural organization of SpNOX and FAD binding

a, Cartoon topology model of SpNOX with the lipid bilayer indicated in grey. **b,** Surface representation of SpNOX showing the electrostatic potential with the positively charged FAD-binding site indicated. k, Boltzmann constant; T, temperature (K); e, charge units. **c,** Schematic representation of FAD binding in SpNOX. **d-f,** The geometry of FAD in SpNOX (**d**), NOX2 (PDB: 8GZ3)[2] (**e**) and human DUOX1 (PDB: 7D3F)[3] (**f**) is conserved, but in DUOX1 it involves positively charged amino acids at the TM domain (Arg1131 and Arg1214) instead of an aromatic residue (Tyr122 in SpNOX; Phe202 in NOX2).

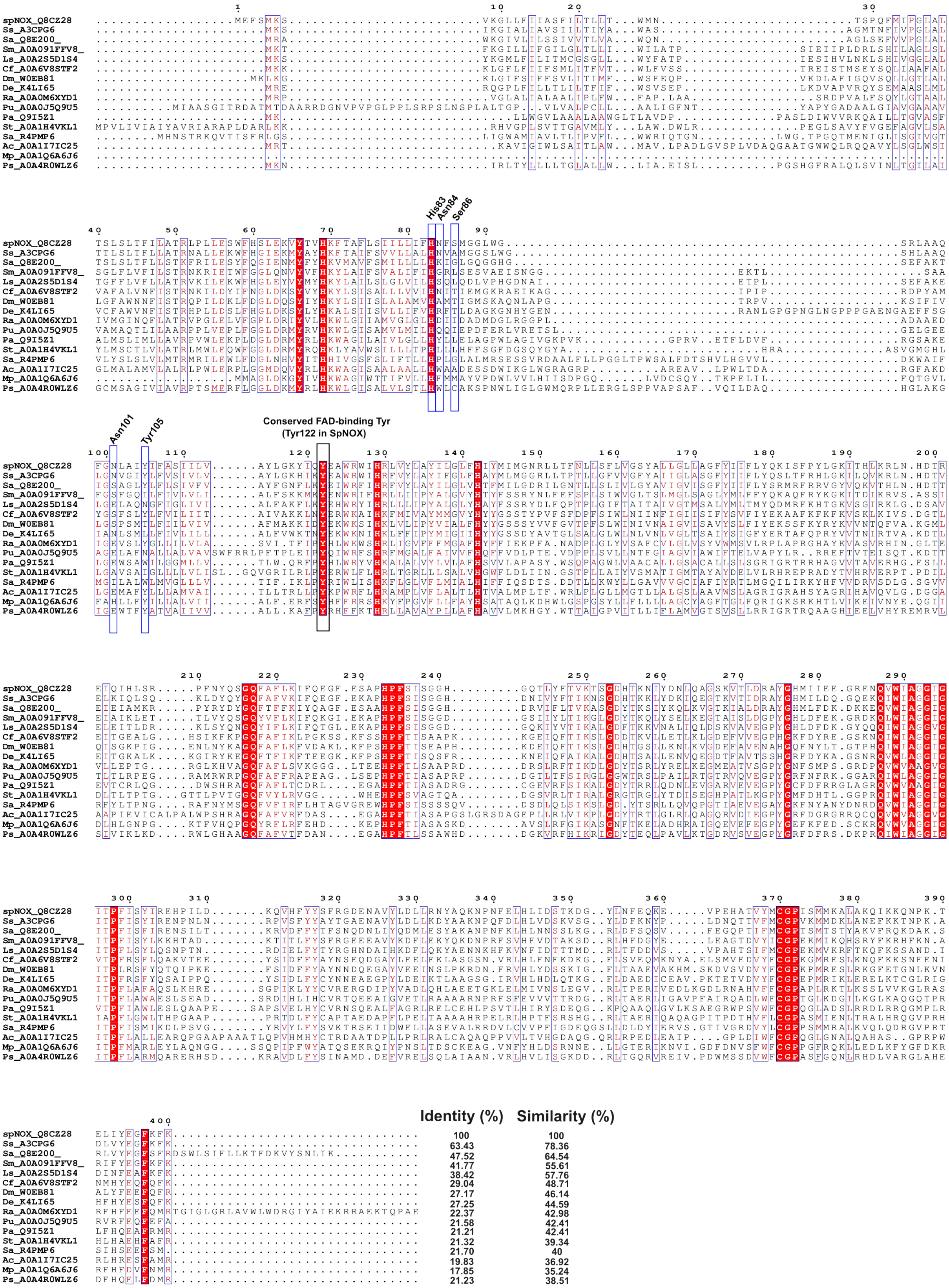

Supplementary Fig. 11 | Sequence alignment of bacterial SpNOX homologues.

The indicated sequence identity is calculated with respect to the spNOX sequence. The amino acids of the putative reaction centers are boxed in blue. The conserved Tyr for binding FAD (Tyr122 in SpNOX) is boxed in black. **De,** *Dehalobacter sp.*; **Cf,** *Clostridium fungisolvens*; **Dm,** *Desulfitobacterium metallireducens*; **Sa,** *Streptococcus agalactiae*; **spNOX,** *Streptococus penumoniae* NOX; **Ss,** *Streptococcus sanguinis*; **Sm,** *Smithella sp.*; **Ls,** *Lysinibacillus sphaericus*; **Sa,** *Saccharimonas aalborgensis*; **Ra,** *Roseibium aggregatum*; **Pu,** *Puniceibacterium sp.*; **St,** *Streptomyces sp.*; **Mp,** *Mucilaginibacter polytrichastri*; **Ps,** *Paraburkholderia strydomiana*; **Pa,** *Pseudomonas aeruginosa*; **Ac,** *Acidovorans caeni*.

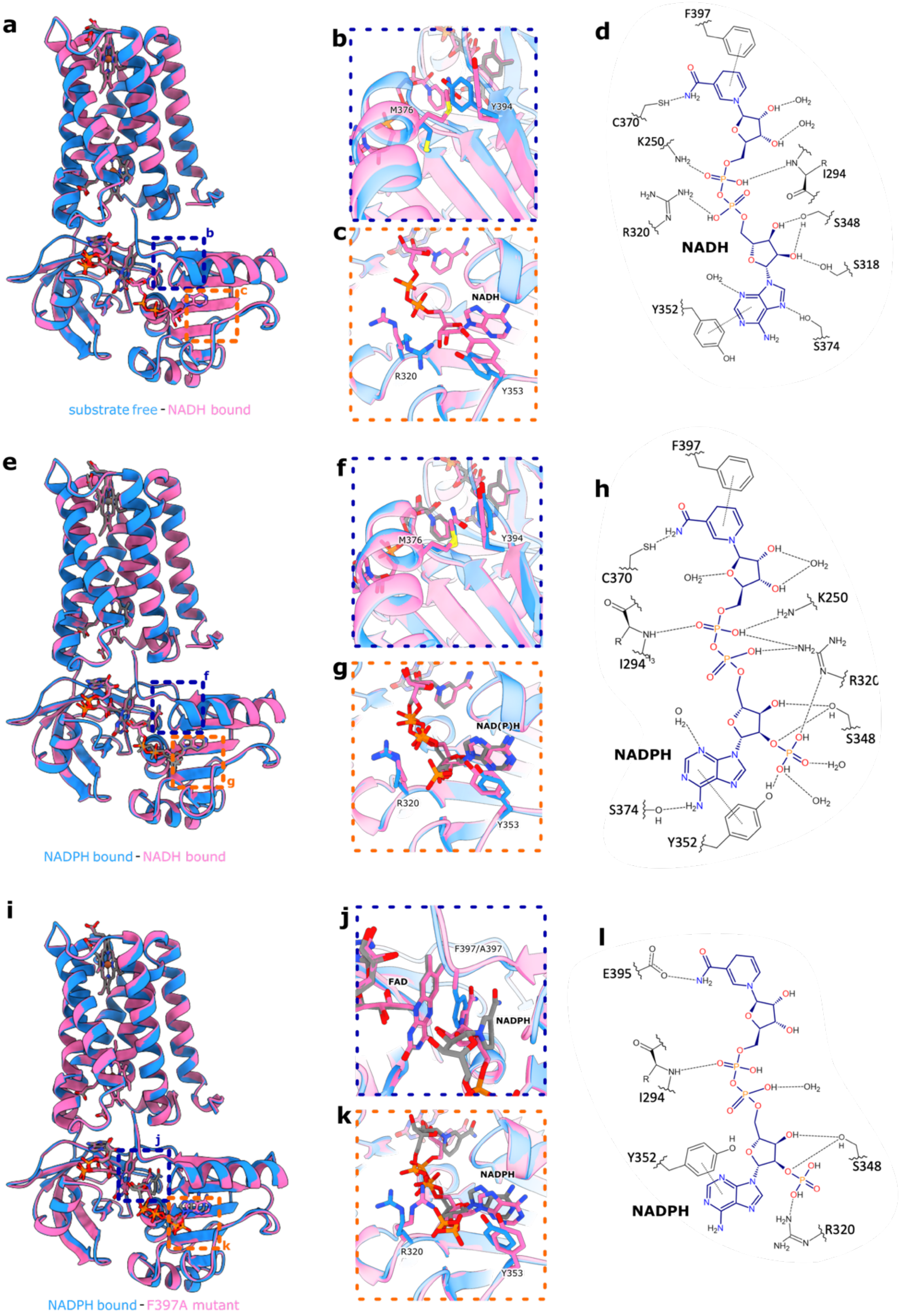

Supplementary Fig. 12 | Comparison between the SpNOX structures

a, Overlayed cartoon models of substrate-free and NADH-bound SpNOX. **b & c,** A closer look at side chain rearrangements upon substrate binding. **d,** Schematic representation of NADH binding. **e,** Overlayed cartoon models of NADPH-bound and NADH-bound SpNOX. **f & g,** A closer look at side chain rearrangements upon substrate binding. **h,** Schematic representation of NADPH binding. **i,** Overlayed cartoon models of NADPH-bound and F397A SpNOX. **j & k,** A closer look at side chain rearrangements upon substrate binding. **l,** Schematic representation of NADPH binding.

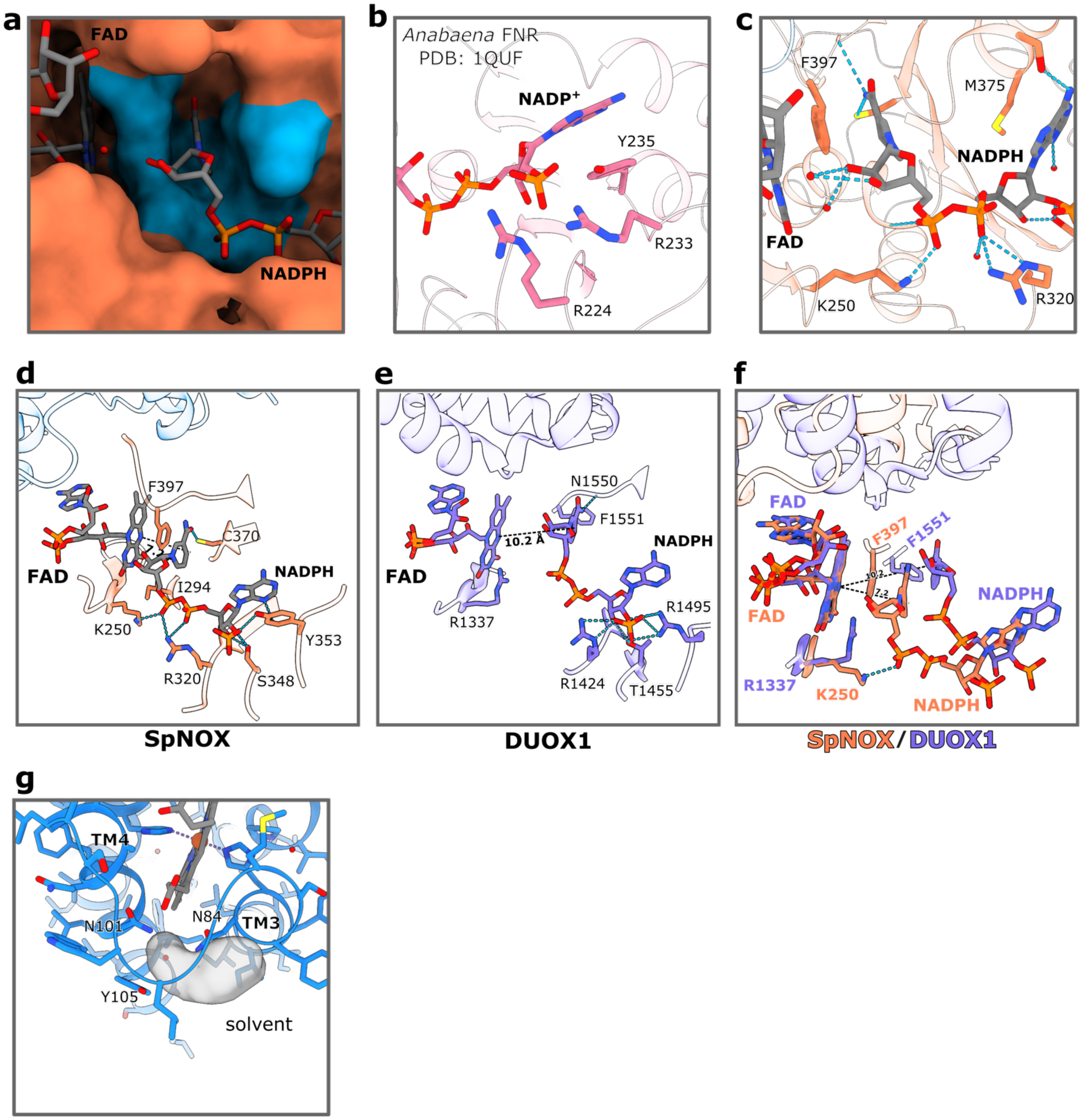

Supplementary Fig. 13 | A detailed view of substrate binding in SpNOX

a, Surface representation of SpNOX with NADPH bound showing the cavity to accommodate nicotinamide formed by the amino acids of the consensus sequences 'XGXGX' and 'CG(S/P)', and by Phe397 (all colored cyan). **b,** Substrate binding of *Anabaena* FNR (PDB: 1QUF) [4] showing the polar interactions between the protein and the 2'-phosphate via arginine residues. **c,** A closer look at the nicotinamide-bound ribose of NADPH reveals the absence of direct interactions with SpNOX. **d-f,** SpNOX achieves a shorter distance between NADPH and FAD (**d**) than high-calcium human DUOX1 (PDB: 7D3F) [3], **e**) via interactions with K250 and F397, which are equivalent to human DUOX1 R1337 and F1551 (**f**). Atoms within H-bond distance are marked with cyan dashed lines. Amino acid side chains are shown as sticks. Only NADPH-interacting amino acids of the DH domains are displayed for simplicity. **g,** A potential $O_2/O_2^{\bullet -}$ entrance and exit path at one of the proposed oxygen binding sites was mapped using the Hollow tool.

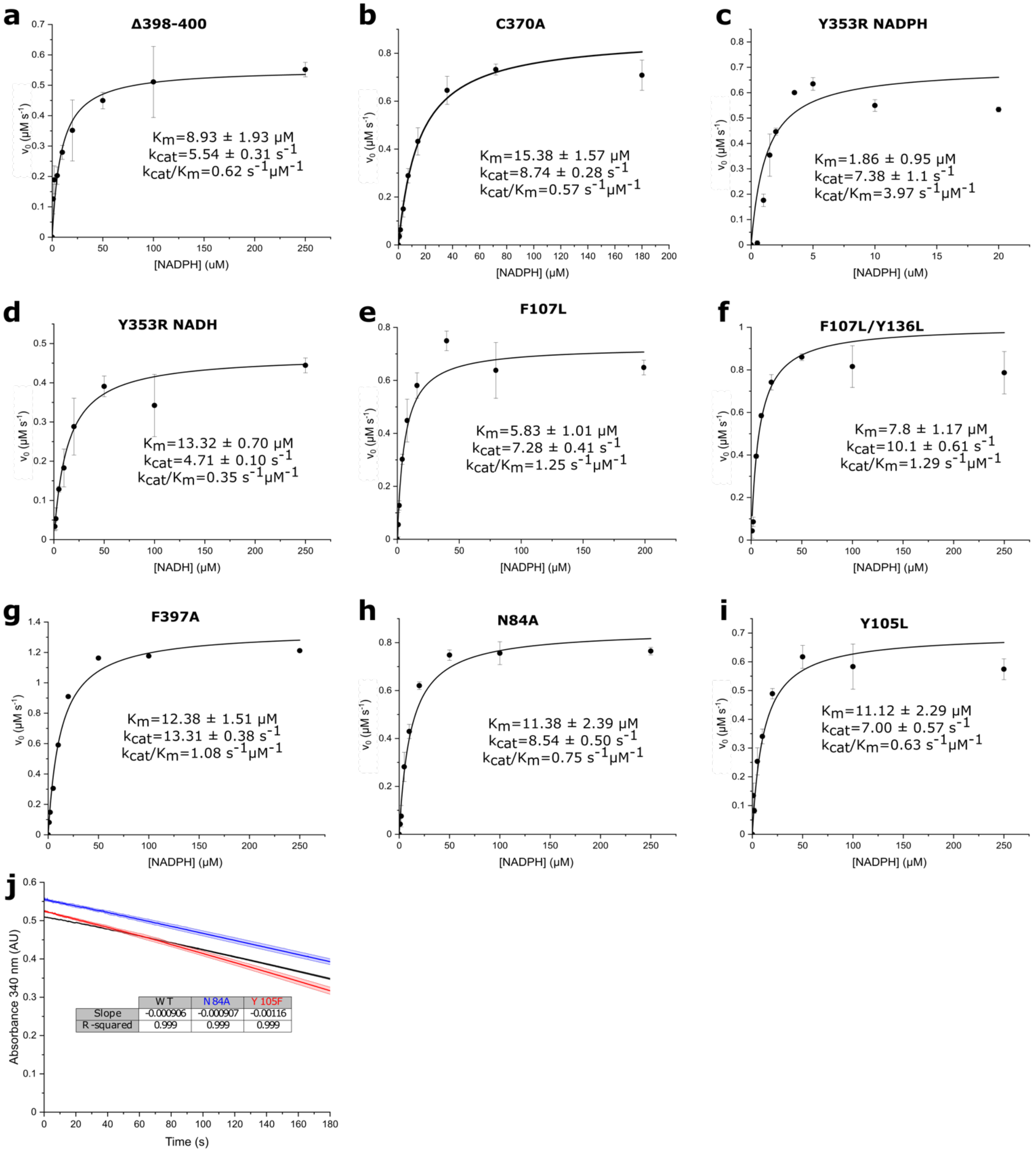

Supplementary Fig. 14 | The NAD(P)H-oxidase activity of SpNOX mutants under steady state conditions. **a-i,** Data obtained from a cytochrome c reduction assay was fitted to the Michaelis-Menten equation to obtain apparent K_m and k_{cat} values. **j,** Data obtained from NADPH oxidation assay. A linear function was fit to obtain the slopes. The NADPH oxidation rates (µM s^{-1}) are calculated using an NADPH extinction coefficient of 6.22 mM^{-1} cm^{-1}. Mean values of three technical replicates (six technical replicates for panel c) are plotted and SD is indicated. Data for individual replicates are available in the source data.

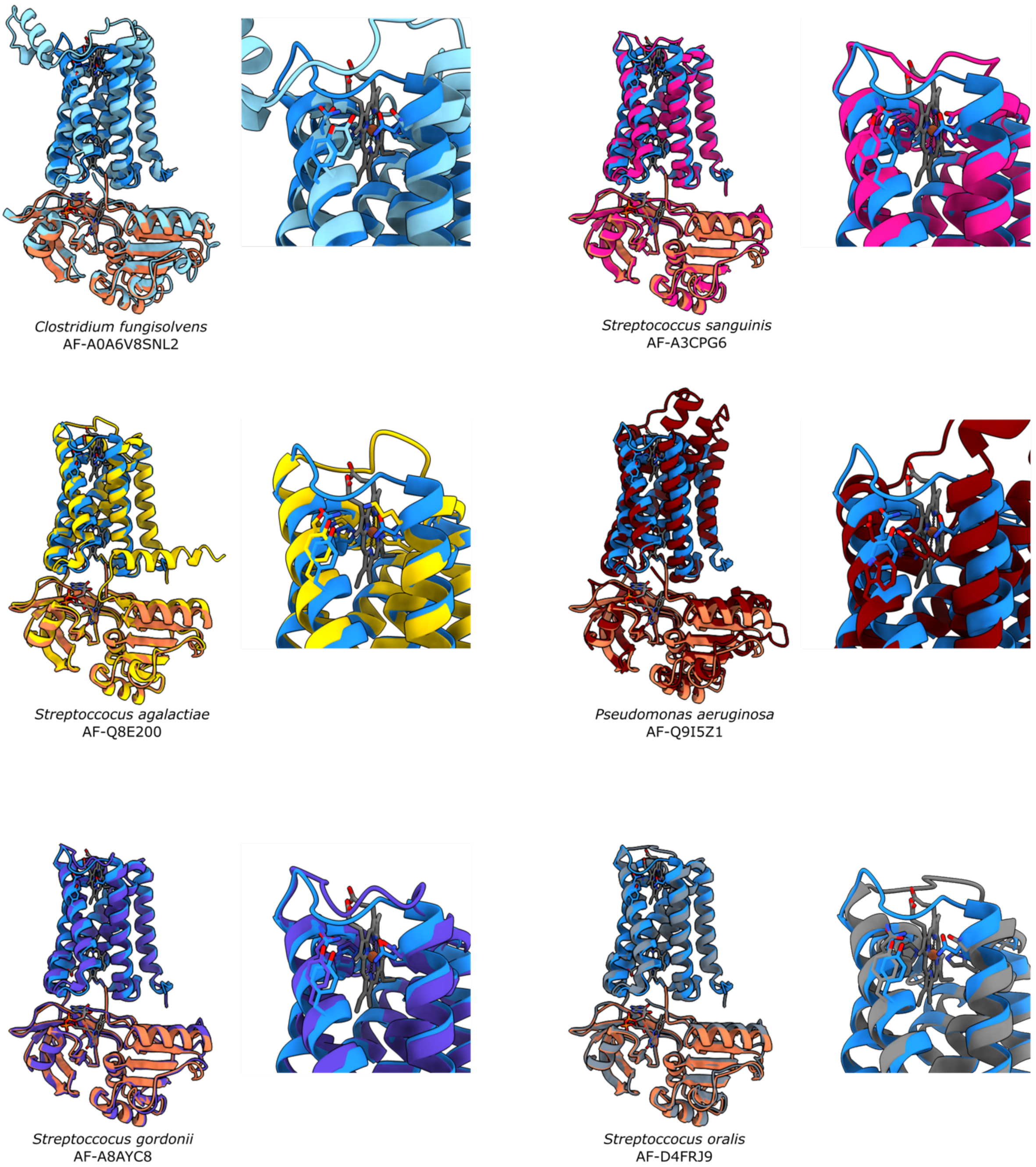

Supplementary Fig. 15 | Predicted structures of bacterial SpNOX-like protein generated by AlphaFold2 showing residue conservation near the outer heme, highlighted in Supplementary Fig. 11.
AlphaFold Protein Structure Database accession codes are indicated under each model.

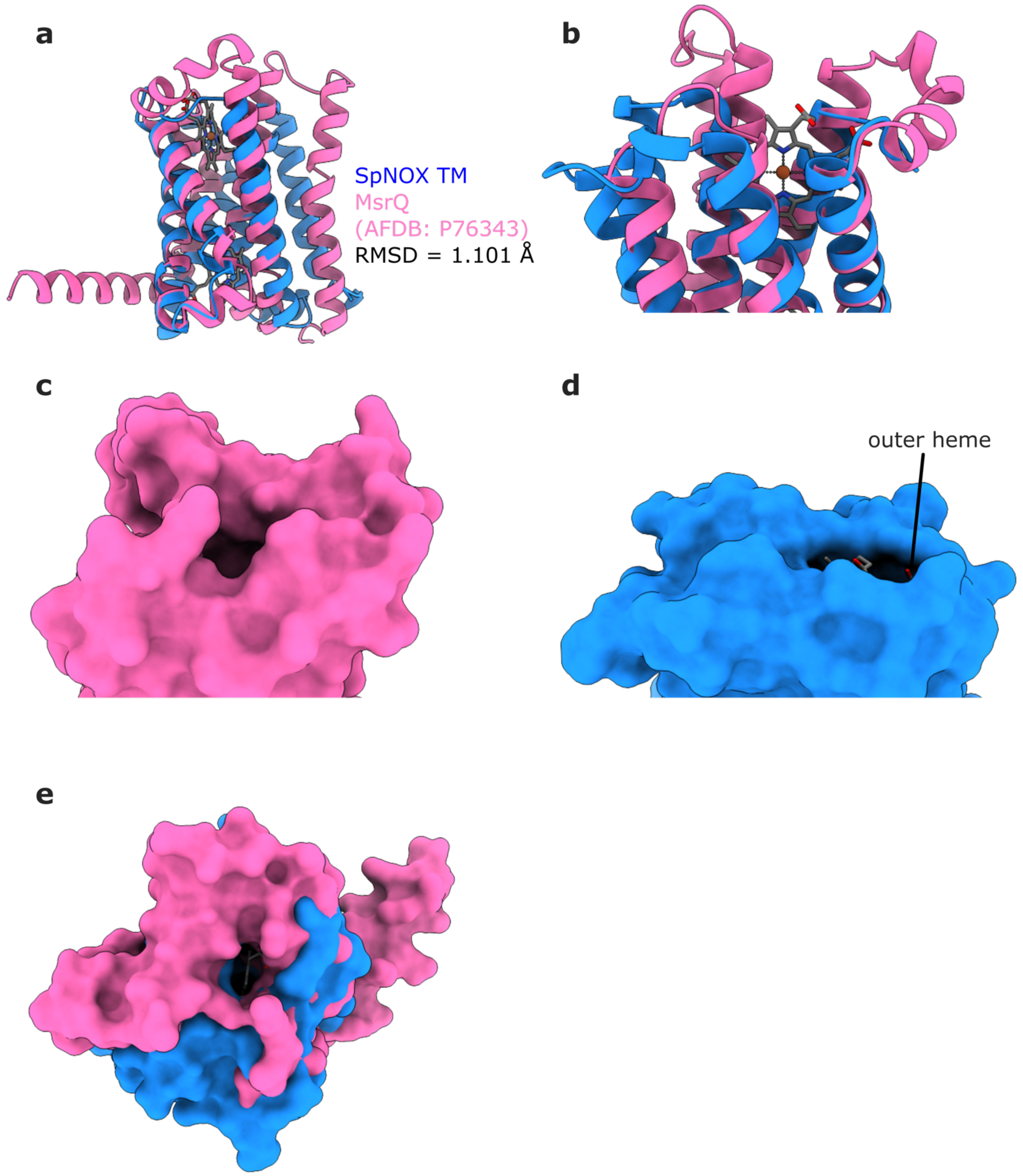

Supplementary Fig. 16 | SpNOX shows an exposed outer heme similar to its homologue MsrQ
a, Cartoon representation of the AlphaFold model of MsrQ (AlphaFold DB: P76343) aligned to SpNOX TMD. RMSD value is given for 38 residue pairs. **b,** A closer look at the periplasmic region of SpNOX and MsrQ. **c,** Surface representation of MsrQ extracellular region showing a large cavity. **d,** Surface representation of SpNOX periplasmic region showing the solvent- accessible outer heme. **e,** Top view of the overlayed surface representations of SpNOX and MsrQ.

Supplementary Table 1. Oligonucleotides used to obtain the fragments for the NEBuilder Hifi Assembly reactions to produce the mutants.

Oligonucleotide	Sequence
**Vector_Rv**	ATTGAAAATTCGGATCCCAGGGGCCCCTGGAACAG
**Insert_Fw**	CCCTGGGATCCGAATTTTCAAT
**F397A_insert_Rv**	cgcACCCTCGTAAATCAGTTCCG
**F397A_vector_Fw**	AACTGATTTACGAGGGTGCGAAGTTCAAGTAAGAATTCGAGCTCCGTCGACAAGCTTG
**Y353R_vector_Fw**	CGTCTGAACTTTGAGCAGAAAGAAGTTCCGGAACACGCG
**Y353R_insert_Rv**	ttctGCTCAAAGTTCAGACGACCGTCCTTGGTGCTGTC
**C370A _vector_Fw**	GCGACGGTCTACATGgcgGGTCCGATTAGCATGATGAAAGCACTGGCGAAACAG
**C370A_insert_Rv**	cgcCATGTAGACCGTCGCGTGTT
**Δ398-400_vector_Fw**	AACTGATTTACGAGGGTttcTAAGAATTCGAGCTCCGTCGACAAGCTTGcggc
**Δ398-400_insert_Rv**	GAAACCCTCGTAAATCAGTTCCG
**Insert_Rv**	TTACTTGAACTTGAAACCCTCGTAAATCAG
**Vector_Fw**	GagGGTTTCAAGTTCAAGTAAGAATTCGAGCTCCGTCGACAAGC
**F107L_insert1_Rv**	aagGATGTAAATCGCCAGATTACCAAACTG
**F107L_insert2_Fw**	ggTAATCTGGCGATTTACATCcttGCTTCCATCATCCTGG
**N84A_insert1_Rv**	GCCGCCCATGCTAAACGCGTGGAAGATCAACAGGATG
**N84A_insert2_Fw**	GAGTTTAGCATGGGCGGCCTGT
**Y105F_insert1_Rv**	Aaa AAT CGC CAG ATT ACC AAA CTG AGC
**Y105F_insert2_Fw**	GGT AAT CTG GCG ATT ttt ATCTTCGCTTCCATCATCCTGG
**F107L/Y136L_insert1_Rv**	AAGCGCCAAGTACACCAGACGAT
**F107L/Y136L_insert2_Fw**	ATCGTCTGGTGTACTTGGCGCTTATCCTTGGTCTGTTCC

Supplementary Table 2. Oligonucleotide combinations used in PCR to obtain the DNA fragments for the NEBuilder HiFi DNA Assembly reactions to generate SpNOX mutants. A pET28a vector with WT SpNOX between NcoI and EcoRI sites was used as template in all reactions except for the F107L/Y136L double mutant, for which the F107L-mutant vector was used instead.

Mutant	Insert1		Insert2		Linearized vector	
	Insert1_Fw	***Insert1_Rv***	***Insert2_Fw***	***Insert2_Rv***	***Vector_Fw***	***Vector_Rv***
F397A	Insert_Fw	F397A_insert_Rv	-	-	F397A_Vector_Fw	Vector_Rv
Y353R	Insert_Fw	Y353R_insert_Rv	-	-	Y353R_vector_Fw	Vector_Rv
C370A	Insert_Fw	C370A_insert_Rv	-	-	C370A _vector_Fw	Vector_Rv
Δ398-400	Insert_Fw	Δ398-400_insert_Rv	-	-	Δ398-400_vector_Fw	Vector_Rv
F107L	Insert_Fw	F107A_insert1_Rv	F107L_insert2_Fw	Insert_Rv	Vector_Fw	Vector_Rv
N84A	Insert_Fw	N84A_insert1_Rv	N84A_insert2_Fw	Insert_Rv	Vector_Fw	Vector_Rv
Y105F	Insert_Fw	Y105F_insert1_Rv	Y105F_insert2_Fw	Insert_Rv	Vector_Fw	Vector_Rv
F107L/Y136L	Insert_Fw	Y136L_insert1_Rv	Y136L_insert2_Fw	Insert_Rv	Vector_Fw	Vector_Rv

References

1. Magnani, F., et al., *Crystal structures and atomic model of NADPH oxidase.* Proceedings of the National Academy of Sciences, 2017. **114**(26): p. 6764-6769.
2. Liu, R., et al., *Structure of human phagocyte NADPH oxidase in the resting state.* eLife, 2022. **11**: p. e83743.
3. Wu, J.-X., et al., *Structures of human dual oxidase 1 complex in low-calcium and high-calcium states.* Nature Communications, 2021. **12**(1): p. 155.
4. Serre, L., et al., *X-ray Structure of the Ferredoxin:NADP+Reductase from the CyanobacteriumAnabaenaPCC 7119 at 1.8 Å Resolution, and Crystallographic Studies of NADP+Binding at 2.25 Å Resolution.* Journal of Molecular Biology, 1996. **263**(1): p. 20-39.

Source data SDS-PAGE gel Supplementary Fig. 1a

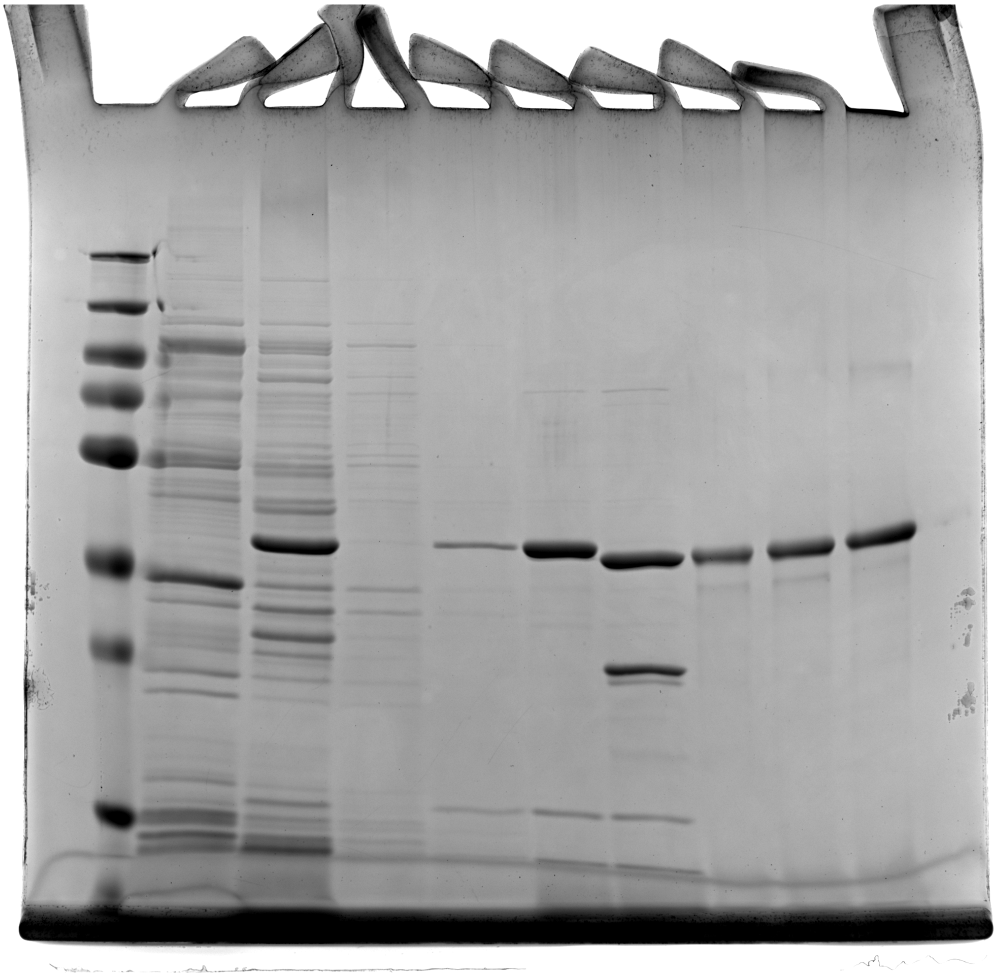

Supplement: Supplementary file 1 — Supplementary Figs. 1–16, Tables 1 and 2, references and uncropped SDS–PAGE gel image. [file 41594_2024_1348_MOESM1_ESM.pdf]
